# PROBIOTIC SUPPLEMENTATION ATTENUATES BINGE EATING AND FOOD ADDICTION 1 YEAR AFTER ROUX-EN-Y GASTRIC BYPASS: A RANDOMIZED, DOUBLE-BLIND, PLACEBO-CONTROLLED TRIAL

**DOI:** 10.1590/0102-672020210002e1659

**Published:** 2022-06-24

**Authors:** Ligia de Oliveira CARLOS, Marilia Rizzon Zaparolli RAMOS, Nathalia Ramori Farinha WAGNER, Lineu Alberto Cavazani de FREITAS, Ingrid FELICIDADE, Antonio Carlos Ligocki CAMPOS

**Affiliations:** 1 Universidade Federal do Paraná, Departamento de Clínica Cirúrgica - Curitiba - PR - Brazil;; 2 Universidade Federal do Paraná, Departamento de Informática - Curitiba - PR - Brazil;; 3 Universidade Estadual de Londrina, Departamento de Biologia Geral - Londrina - PR - Brazil.

**Keywords:** Probiotics, Bariatric Surgery, Binge-Eating Disorder, Food Addiction, Probióticos, Cirurgia Bariátrica, Transtorno da Compulsão Alimentar, Dependência de Alimentos

## Abstract

The use of probiotics as adjuvants in the treatment of eating disorders, known as psychobiotics, has already been investigated as a means of modulating the microbiota-gut-brain axis.

**AIM:**

This study aimed to assess the effect of probiotic supplementation on binge eating and food addiction in subjects after Roux-en-Y gastric bypass surgery.

**METHODS:**

This is a randomized, double-blind, placebo-controlled trial involving 101 patients who received probiotic (*Lactobacillus acidophilus* NCFM and *Bifidobacterium lactis* Bi-07) or placebo supplements for 90 days after bariatric surgery, starting on the seventh postoperative day. They were evaluated preoperatively (T0) and postoperatively at 90 days (T1) and 1 year (T2) after surgery. The Yale Food Addiction Scale (YFAS) and Binge Eating Scale (BES) were applied to assess food addiction and binge eating, respectively.

**RESULTS:**

Before surgery, one-third of the patients presented with a food addiction and binge eating diagnosis. The number of symptoms of YFAS and the BES score decreased significantly in both groups at T1 compared to T0. However, a significant effect of treatment with probiotics was observed 1 year after surgery (T2). Both the number of symptoms of food addiction and the binge eating score were lower in the probiotic group than in the placebo group (p=0.037 and p=0.030, respectively).

**CONCLUSION:**

The use of probiotic supplementation for 90 days in the immediate postoperative period may decrease food addiction symptoms and binge eating score up to 1 year after surgery compared to controls.

## RESUMO - RACIONAL

O uso de probióticos como coadjuvantes no tratamento de distúrbios alimentares, conhecidos como psicobióticos, já foi investigado na modulação do eixo intestino-microbiota-cérebro.

**OBJETIVO:**

Analisar a influência da suplementação com probióticos no vício e compulsão alimentar em indivíduos submetidos à cirurgia de bypass gástrico em Y-de-Roux

**MÉTODOS:**

Trata-se de um estudo randomizado, duplo-cego, controlado por placebo, envolvendo 101 pacientes que receberam suplementação de probiótico (*Lactobacillus acidophilus* NCFM e *Bifidobacterium lactis* Bi-07) ou placebo, durante 90 dias após a cirurgia bariátrica, com início no sétimo dia de pós-operatório. Os indivíduos foram avaliados no pré-operatório (T0) e no pós-operatório aos 90 dias (T1) e 1 ano (T2) após a cirurgia. A Escala de Dependência Alimentar de Yale (YFAS) e a Escala de Compulsão Alimentar Periódica (ECAP) foram aplicadas para avaliar o vício e compulsão alimentar, respectivamente.

**RESULTADOS:**

Antes da cirurgia, um terço dos pacientes apresentou diagnóstico de dependência alimentar e compulsão alimentar. O número de sintomas da YFAS e a pontuação da ECAP diminuiu significativamente em ambos os grupos em T1 em comparação com T0. Entretanto, um ano após a cirurgia (T2), tanto o número de sintomas de vício alimentar como a pontuação de compulsão alimentar foram menores no grupo probiótico do que no grupo placebo (p = 0,037 e p = 0,030, respectivamente).

**CONCLUSÃO:**

A utilização de suplemento probiótico durante 90 dias após a cirurgia pode diminuir os sintomas de vício alimentar e a pontuação de compulsão alimentar um ano após a cirurgia em comparação com o grupo controle.

## INTRODUCTION

Obesity remains a threat to global health due to a heterogeneous condition with clinical needs that are still largely unsatisfactory. Worldwide, overweight and obesity have nearly tripled in the past four decades. It is estimated that at least 2.8 million people die each year as a result ^26^ ^,^ ^48^.

In severe obesity, bariatric surgery is considered the gold standard for treatment, achieving consistent results in inducing change in body mass index (BMI), remission of comorbidities, and improving quality of life^26^, though postbariatric surgery patients generally maintain substantial % total weight loss (%TWL), weight regain occurs. It was estimated that an average of 23.8% of the %TWL recovered in the first 6 years after surgery^20^. Diverse and overlapping factors are proposed to explain weight regain, including problematic eating behaviors^19^ ^,^ ^21^ ^,^ ^24^ ^,^ ^25^.

Eating disorders are serious psychiatric illnesses characterized by abnormal eating behavior and/or excessive preoccupation with body weight^42^. The prevalence of eating disorders among individuals undergoing bariatric surgery varies according to the assessment method, but it seems to be higher than that in the general population^41^ ^,^ ^43^. Moreover, binge eating disorder (BED) is the second most common single disorder in bariatric patients^30^.

BED is recognized as a distinct eating disorder in the *Diagnostic and Statistical Manual of Mental Disorders* (*DSM*)^1^ and is characterized by the consumption of large amounts of food in a short period of time and a sense of loss of control over eating during these episodes. BED is associated with distress and regret for the individual^46^.

Recently, researchers have investigated “food addiction” (FA) among individuals undergoing bariatric surgery^17^. Energy-dense food are generally rich in sugar, fat, and/or salt and are consequently very palatable. These foods are excessively stimulating for the reward pathways of the brain that can promote craving, an uncontrollable urge, an insatiable desire to continue eating and trigger symptoms, but still not so associated with abstinence^3^ ^,^ ^36^. Although the *DSM-5* criteria have not recognized FA as a viable addiction, several authors are conducting studies in this context^3^ ^,^ ^4^ ^,^ ^5^. Brewerton^5^ associated the severity of FA with BED and its combination with psychopathologies and a greater severity of obesity.

Unfortunately, there are significant limitations in the ability to detect, prevent, and treat these disorders. The classic treatment for eating disorders is psychotherapy that may or may not be combined with medication^5^ ^,^ ^10^ ^,^ ^43^. A new approach for treating psychiatric disorders is the use of probiotics and prebiotics as modulators of the microbiota-gut-brain axis, also known as psychobiotics^23^ ^,^ ^27^. Recently, studies have investigated the effects of the use of psychobiotics in patients with depression, anxiety, and obesity^44^.

Furthermore, studies using supplementation with probiotics in individuals undergoing bariatric surgery were conducted to verify their effects on %TWL, quality of life, and gastrointestinal discomfort^9^ ^,^ ^40^.

However, studies evaluating the influence of probiotic supplementation on psychological or behavioral factors in individuals undergoing bariatric surgery are still lacking^9^. Therefore, the aim of this study was to analyze the influence of probiotic supplementation on BED and FA in individuals undergoing Roux-en-Y gastric bypass (RYGB).

## METHODS

### Experimental Design

This is a randomized, double-blind, placebo-controlled clinical trial conducted with patients undergoing RYGB from April 2018 to December 2019. The study was approved by the Research Ethics Committee (nº 2.810.276 - clinical trial nº RBR-4x3gqp). The research was explained to each participant prior to their participation, and informed written consent was obtained from those who agreed to participate.

The randomization of the samples was performed according to the protocol disclosed in a previously published paper with clinical and metabolic data of a similar cohort of patients^32^. Briefly, the inclusion criteria were as follows: adults (18-59 years old) who would be submitted to RYGB, had a BMI=35 kg/m^2^, and did not use antibiotics in the 4 weeks prior to surgery. Patients who underwent other surgical techniques or reoperation, had postsurgical complications, had antibiotic therapy concomitant with probiotic/placebo supplement use, or did not use probiotic/placebo tablets for more than 9 consecutive days (adherence less than 90%) were withdrawn from the study. The researchers randomized the individuals by a systematic 1:1 allocation process.

On the seventh postoperative day, the participants were instructed to ingest two chewable tablets/day of either a placebo, an inert manipulated tablet, or a probiotic tablet (Flora Vantage, 5 billion *Lactobacillus acidophilus* NCFM^®^ Strain and 5 billion *Bifidobacterium lactis* Bi-07^®^) from Bariatric Advantage (Aliso Viejo, CA, USA) for 90 days.

Both groups received the same dietary orientations after surgery, were followed by the same surgical team (i.e., doctor, dietitian, and psychologist), and had the same number of prescheduled appointments before and after surgery, following the protocol established by the Institution where the study was carried out.

### Data Collection

The first assessment (T0) was performed approximately 10 days before surgery. Follow-up assessments were conducted approximately 3 months (T1) and 1 year postoperatively (T2). Clinical and anthropometric assessments were performed, and self-administered questionnaires were administered to the participants at every meeting. Anthropometric measurements included body weight (kg), height (m), and BMI (kg/m^2^)^47^.

### Questionnaires

####  Binge Eating 

The Binge Eating Scale (BES) is a 16-item self-report measure created by Gommarly et al^16^ and translated and adapted into Portuguese by Freitas et al^13^. The BES is a tested and reliable instrument, and it remains one of the most commonly used screening tools for measuring binge eating. The BES has been employed in multiple studies with bariatric patients before and after the procedure^22^ ^,^ ^29^.

Individuals were instructed to select the answer that best represented their response, and the final scores were obtained by (1) the BES total score: the sum of the points of each item (ranging from 0 to 46), thus measuring the binge eating severity, and (2) the binge eating severity classification (according to the BES total score) as follows: (1) score=17: none; (2) score=18-26: moderate; and (3)score=27: severe binge eating.

####  Food Addiction 

The Yale Food Addiction Scale (YFAS), a self-report questionnaire that detects symptoms of addictive eating behaviors, was used to assess FA.

The YFAS was based on the DSM-IV-Text Revision substance dependence criteria and endorsed for highly processed foods^2^. This questionnaire was developed by Gearhardt et al^15^ and validated for Portuguese by Torres et al^42^ among patients after bariatric surgery^8^.

The questionnaire is a combination of 25 Likert and dichotomous scoring options that provides two assessment options: (1) FA “‘diagnosis” = three “symptoms” are present, and a clinically significant impairment or distress is endorsed and (2) number of FA “”symptoms.” The symptoms are described in Supplementary material.

### Statistical Analysis

The characteristics of the sample are presented as mean±standard deviation for continuous variables and as a percentage for categorical variables. The statistical analyses were carried out using the R software^34^. Shapiro-Wilk tests were performed to assess the normality distribution of continuous variables, and the Mann-Whitney U test was used to assess the difference between placebo and probiotic groups in continuous variables.

We used generalized additive models for location scale and shape (GAMLSS)^39^ to evaluate the effect of probiotic use on the response variables (i.e., FA and BED) and on other explanatory variables (i.e., age, body weight, and BMI) for the same individual over the analyzed period.

GAMLSS are a distributional regression^33^ approach that extends the well-known generalized linear models (GLMs) and generalized additive models (GAMs) that have mechanisms to support characteristics that must be considered in the analysis process, such as measurements taken from the same individual over time when normal distribution does not apply.

## RESULTS

### Characteristics of the Participants

**Figure 1 -:**
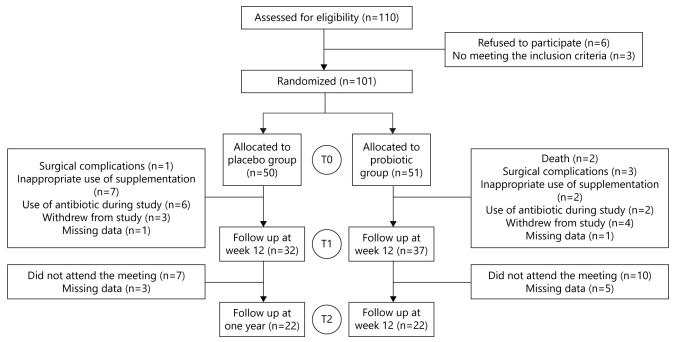
The study consort flowchart. T0, presurgical moment; T1, 3 months postsurgical moment; T2, 1 year postsurgical moment.

Out of 110 patients initially selected, 70.3% completed the supplementation protocol, and 44 were followed up for 1 year after RYGB surgery (Figure 1).

Most of the patients were female (87.30%), with an average age of 40 (±11.25) years old. Participants were randomized in the placebo and probiotic groups and had 99% adherence to supplementation in both groups. None of the participants reported adverse effects during the intervention. Anthropometric and eating behavior data of individuals before and after RYGB are described in Table 1 and show the similarity of the groups regarding anthropometry at all time points. Body weight, BMI, and age seemed to have no impact on the results.

**Table 1 -:** Anthropometric and eating behavior data of individuals before and after Roux-en-Y gastric bypass.

	T0			T1			T2		
	CG (n=33)	PG (n=38)	p	CG (n=32)	PG (n=37)	p	CG (n=22)	PG (n=22)	p
Weight (kg)	111.21 ± 17.57	113.61 ± 23.21	0.95	87.72 ± 13.81	91.31 ± 19.21	0.71	74.39 ± 13.34	73.18 ± 21.12	0.34
IMC (kg/m^2^)	43.51 ± 5.51	42.84 ± 5.40	0.52	34.59 ± 4.68	34.79 ± 5.15	0.71	28.75 ± 4.33	27.94 ± 5.33	0.42
Food addiction									
FA (yes)	6 (18.18)	17 (44.73)	NA	1 (3.12)	1 (2.70)	NA	0 (0)	0 (0)	NA
N° of symptoms	2.94 ±2.01	3.89±1.9	0.025	0.87±1.24	0.70 ± 0.97	0.076	1.27 ± 1.16	0.82 ± 1.01	0.141
Binge eating									
No BED	25 (75.75)	23 (60.52)	NA	31 (96.87)	37 (100)	NA	21 (95.45)	22 (100)	NA
Moderate BED	4 (12.12)	11 (28.95)	NA	1 (3.12)	0 (0)	NA	1 (4.54)	0 (0)	NA
Severe BED	4 (12.12)	4 (10.53)	NA	0 (0)	0 (0)	NA	0 (0)	0 (0)	NA
BES score	11.21 ± 9.12	14.63 ± 8.17	0.043	4.25±4.56	4.38 ± 3.57	0.572	4.77 ± 5.54	3.13 ± 4.28	0.360

T0, presurgical moment; T1, 3 months postsurgical moment; T2, 1 year postsurgical moment; CG, placebo group; PG, probiotic group; YFAS, Yale Food Addiction Scale; FA, food addiction; BED, binge eating disorder; BES, Binge Eating Scale; NA, not applicable. Quantitative variables are expressed as mean and standard deviation (±SD); qualitative variables as percentage or n (%). A p-value between groups was obtained with Mann-Whitney U test. Because the frequency of data is low, no statistical test can be used to compare the groups.

**Figure 2 -:**
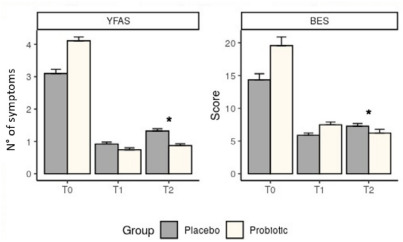
Predictive number of symptoms of food addiction and Binge Eating Scale (BES) of individuals in the presurgical moment and postsurgical of 3 months and 1 year of Roux-en-Y gastric bypass. T0, presurgical moment; T1, 3 months postsurgical moment; T2, 1 year postsurgical moment; YFAS, Yale food addiction scale; FA, food addiction; . *Statistical difference between groups. Generalized additive models for location scale and shape (GAMLSS) was used as statistical analyses.

Randomly, YFAS symptoms and BES scores were higher at the T0 time point in the probiotic group than in the control. However, there was a decrease in YFAS and BES at T1 compared to T0 in both groups and a trend toward an increase in these values at T2 compared to T1 (Figure 2). The probiotic group behaved differently from the placebo group at T2, with a lower increase in the number of YFAS and a continued decrease in BES values. These results highlight the impact of probiotic supplementation; even though this group had higher values before the intervention (T0), at T2, they had fewer YFAS symptoms and a lower BES score.

Table 2 presents the GAMLSS results. There was a significant effect of probiotic treatment observed 1 year after surgery compared to the placebo group in both YFAS and BES (p=0.037 and p=0.030, respectively), which was not the case at T1 (0.076 and 0.674, respectively).

**Table 2 -:** Random-effect models results assessing Yale Food Addiction Scale symptoms and Binge Eating Scale after 3 months and 1 year of Roux-en-Y gastric bypass surgery.

	YFAS symptoms		BES	
Fixed effects	Coefficient (SE)	p-value	Coefficient (SE)	p-value
Intercept	1.13 (0.102)	<0.001	2.664 (0.069)	<0.001
T1	-1.21 (0.183)	<0.001	-0.893 (0.123)	<0.001
T2	-0.851 (0.231)	<0.001	-0.682 (0.139)	<0.001
Probiotic	0.284 (0.15)	0.060	0.310 (0.089)	0.001
T1*Probiotic	-0.499 (0.28)	0.076	-0.069 (0.164)	0.674
T2*Probiotic	-0.698 (0.332)	0.037	-0.464 (0.211)	0.030

YFAS, Yale Food addiction scale; BES, Binge Eating Scale; T0, presurgical moment; T1, 3 months postsurgical moment; T2, 1 year postsurgical moment; SE, standard error. Generalized additive.

## DISCUSSION

For the first time, early probiotic supplementation was given to individuals who underwent RYGB surgery with the aim of verifying the impact on FA and binge eating 3 months and 1 year after bariatric surgery. Probiotic supplementation was associated with an attenuation of the binge eating score and FA symptoms 1 year after bariatric surgery.

The present study included a population with characteristics similar to those of general bariatric individuals^14^: predominantly female (87.3%), a mean age of 40.21 years, and a mean BMI of 43.16 kg/m^2^. We observed a similar average BMI for the groups at all time points evaluated (i.e., T0, T1, and T2), and these covariables (i.e., weight body, BMI, and age) seemed to have no impact on the results. In addition, the presurgical food dependence rate and BES were similar to other research also conducted with obese prebariatric patients^6^ ^,^ ^11^ ^,^ ^22^ ^,^ ^35^. However, in our study, when each group was analyzed, the probiotic group had randomly higher BES and YFAS rates than the placebo group at the presurgical time point.

Previous literature^6^ ^,^ ^21^ ^,^ ^41^ has shown that even though it is important to identify individuals at high risk for eating disorders, it is more important to follow them in the postoperative period and apply appropriate interventions to maximize clinical outcomes, such as reducing psychiatric and somatic complications^21^ and improving quality of life^31^ and %TWL^25^. Our patients were followed up prospectively. Three months after the RYGB surgery, there was a decrease in the mean BES scores and number of FA symptoms, showing that bariatric surgery impacts these parameters. The reasons for these changes probably include the many dietary changes that occur in the first months after surgery, as well as the patient’s fear of having adverse effects if they do not follow the nutritional protocol^21^.

Ben Porat et al^4^ evaluated FA before sleeve surgery and found 40.7% had FA diagnoses, although 3 months later, these rates decreased to 10.2%. Similarly, the diagnosis of BE decreased from 48.1% to 10.2% before and after surgery, respectively. This same research group continued the study and verified an increase in the percentage of people diagnosed with FA and BE 1 year after surgery compared to the 3-month evaluation (29.3% and 17.4%, respectively).

We also observe that YFAS tended to increase at T2 as compared to T1 for both groups. However, only the BES of the placebo group shows this trend, as the probiotic group continued to decline. In our research, the use of the probiotic supplementation after RYGB surgery attenuated YFAS and BES 1 year after the surgery, when these indices start to increase^4^ ^,^ ^35^. Thus, this supplementation could be used as an adjuvant in the treatment of eating disorders after bariatric surgery.

To the best of our knowledge, the strains used for the supplements given to these patients (*Lactobacillus* and *Bifidobacterium*) have not yet been used in patients with FA and BED. However, these results are in accordance with Cook et al^9^, who suggested that some strains of *Lactobacillus* and *Bifidobacterium* may be helpful in controlling long-term obesity and have potential effects on central nervous system function and probable effects on mood, anxiety, and cognition.

Although the focus of this study was not to evaluate the probiotic mechanism of action, it is known that by modifying the gastrointestinal tract microbiome, probiotics may influence the production of substrates that influence various systems that impact the central nervous system and consequently human behavior^27^. The main mechanisms by which probiotics can influence addiction and compulsion are (1) increased production of short-chain fatty acids (hindering lipopolysaccharide produced by pathogenic commensal bacteria, downregulating zonulin, and decreasing paracellular permeability)^18^ ^,^ ^44^; (2) inflammation regulation (decreasing endotoxemia and improving neuroplasticity through brain-derived neurotrophic factor gene expression)^7^; (3) modulation of immune system function^13^ ^,^ ^27^; (4) decreased cortisol production by downregulation of the hypothalamic-pituitary-adrenal (HPA) axis^27^ ^,^ ^37^; (5) pleiotropic effects of enteroendocrine cells^12^; and (6) improvement of serotonin and gamma-aminobutyric acid (GABA) biosynthesis (activating the vagus nervous system)^13^ ^,^ ^18^.

### Strength and limits

The strengths of our study are the design (randomized, double-blind, placebo-controlled), the similarity among groups (same surgical technique and anthropometric data), the use of probiotics and a placebo developed for this specific supplementation period that are both chewable and palatable, the weekly contact between researchers and participants to monitor their adherence to the research protocol, and the high adherence achieved with the use of probiotics or placebo (over 99%). These results also highlight the importance of assessing a range of behaviors rather than only categorical diagnoses^28^ ^,^ ^38^ and the benefits of using adjuvant treatments to improve the results of bariatric surgery.

The main limitation of this study is the lack of gut microbiota analysis and a follow-up rate at 1 year lower than 50%. However, we used regression models of the GAMLSS class to study the effect of timing and probiotic use on addiction and compulsion metrics. Regression models are statistical techniques that allow us to assess the impact of explanatory variables on response variables by estimating quantities that measure this effect. If this quantity measuring the effect is different from 0, there is evidence of a significant effect of the explanatory variable on the response. This evaluation is done using a simple hypothesis test. The results of these hypothesis tests are shown in Table 2, where the most important result is in the last row and shows a significant effect of the probiotic use 1 year after surgery.

## CONCLUSION

The findings demonstrate the ability of early probiotic supplementation may decrease binge eating and symptoms of FA 1 year after RYGB surgery. Future research should also examine the impact of early and late probiotic supplementation on eating disorders, as well as those effects in nonobese and nonbariatric individuals.
